# Mortality and Its Predictors Amongst Patients With Advanced Dementia Receiving Psychiatric Inpatient Care

**DOI:** 10.1002/alz.090699

**Published:** 2025-01-03

**Authors:** Oriane E Marguet, Shanquan Chen, Emad Sidhom, Emma Wolverson, Gregor Russell, George Crowther, Simon R White, Rebecca Dunning, Hasan Shahrin, Benjamin R Underwood, Jonathan Lewis

**Affiliations:** ^1^ University of Cambridge, Cambridge, Cambridgeshire United Kingdom; ^2^ Keppel St, London, London, London United Kingdom; ^3^ Dementia UK, London, London United Kingdom; ^4^ University of Hull, Hull, East Yorkshire United Kingdom; ^5^ Bradford District Care NHS Foundation Trust, Bradford United Kingdom; ^6^ Leeds and York Partnership NHS Foundation Trust, Leeds, West Yorkshire United Kingdom; ^7^ University of Cambridge, Cambridge United Kingdom; ^8^ MRC Biostatistics Unit, University of Cambridge, Cambridge United Kingdom; ^9^ Humber Teaching NHS Foundation Trust, Hull, East Riding of Yorkshire United Kingdom; ^10^ Bradford District Care NHS Foundation Trust, Bradford, West Yorkshire United Kingdom; ^11^ Cambridgeshire and Peterborough NHS Foundation Trust, Cambridge United Kingdom; ^12^ Ambridgeshire and Peterborough NHS Foundation Trust, Cambridge, Cambridgeshire United Kingdom

## Abstract

**Background:**

People with dementia frequently develop behavioural and psychological symptoms, sometimes necessitating care in specialist dementia mental health wards. There has been little research on their life expectancy following admission or need for palliative care. The work presented here explores the mortality of these patients and whether this can be predicted at their time of admission to the ward.

**Method:**

We conducted a retrospective analysis of 576 patients admitted to the Cambridgeshire and Peterborough NHS Foundation Trust dementia mental health wards in the United Kingdom, and built a Kaplan‐Meier survival curve as well as machine learning models. Next, to examine changes in deaths occurring over time, a retrospective service evaluation was conducted involving four mental health wards for people with dementia in the United Kingdom, encompassing a further 1,976 patients.

**Result:**

The median survival length post‐admission was 1201 days. Clinical data collected on admission did not predict mortality in machine learning models at a level of accuracy likely to have clinical utility. Data from four different wards show that the number of patients dying in dementia mental health wards has increased over time.

**Conclusion:**

Our cohort had a high mortality, although with a wide range of survival times. We suggest all people admitted to these units should have discussions and access to high‐quality end‐of‐life care.

